# AKAZE-GMS-PROSAC: A New Progressive Framework for Matching Dynamic Characteristics of Flotation Foam

**DOI:** 10.3390/jimaging12010007

**Published:** 2025-12-25

**Authors:** Zhen Peng, Zhihong Jiang, Pengcheng Zhu, Gaipin Cai, Xiaoyan Luo

**Affiliations:** 1 School of Electrical Engineering and Automation, Jiangxi University of Science and Technology, Ganzhou 341000, China; 6720240789@mail.jxust.edu.cn (Z.P.); 6720240796@mail.jxust.edu.cn (P.Z.); cgp4821@163.com (G.C.); 2 School of Mechanical and Electrical Engineering, Jiangxi University of Science and Technology, Ganzhou 341000, China; 9119960010@jxust.edu.cn; 3 Key Laboratory of Particle Technology of Jiangxi Province, Nanchang 330013, China

**Keywords:** flotation foam image, dynamic characteristic analysis, feature matching, AKAZE-GMS-PROSAC, progressive optimization

## Abstract

The dynamic characteristics of flotation foam, such as velocity and breakage rate, are critical factors that influence mineral separation efficiency. However, challenges inherent in foam images, including weak textures, severe deformations, and motion blur, present significant technical hurdles for dynamic monitoring. These issues lead to a fundamental conflict between the efficiency and accuracy of traditional feature matching algorithms. This paper introduces a novel progressive framework for dynamic feature matching in flotation foam images, termed “stable extraction, efficient coarse screening, and precise matching.” This framework first employs the Accelerated-KAZE (AKAZE) algorithm to extract robust, scale- and rotation-invariant feature points from a non-linear scale-space, effectively addressing the challenge of weak textures. Subsequently, it innovatively incorporates the Grid-based Motion Statistics (GMS) algorithm to perform efficient coarse screening based on motion consistency, rapidly filtering out a large number of obvious mismatches. Finally, the Progressive Sample and Consensus (PROSAC) algorithm is used for precise matching, eliminating the remaining subtle mismatches through progressive sampling and geometric constraints. This framework enables the precise analysis of dynamic foam characteristics, including displacement, velocity, and breakage rate (enhanced by a robust “foam lifetime” mechanism). Comparative experimental results demonstrate that, compared to ORB-GMS-RANSAC (with a Mean Absolute Error, MAE of 1.20 pixels and a Mean Relative Error, MRE of 9.10%) and ORB-RANSAC (MAE: 3.53 pixels, MRE: 27.36%), the proposed framework achieves significantly lower error rates (MAE: 0.23 pixels, MRE: 2.13%). It exhibits exceptional stability and accuracy, particularly in complex scenarios involving low texture and minor displacements. This research provides a high-precision, high-robustness technical solution for the dynamic monitoring and intelligent control of the flotation process.

## 1. Introduction

Mineral resources are the cornerstone of global industrial development, yet the grade of raw ore is generally low. As industrialization progresses, the efficiency of traditional mineral processing techniques is diminishing, particularly for fine-grained minerals. Flotation, as a core process in modern mineral processing, relies heavily on the dynamic characteristics of its foam layer, such as velocity and breakage rate, which play a decisive role in separation efficiency. These two parameters are not independent; rather, they are two equivalent and core indicators for evaluating the overall dynamic state of the foam layer. Velocity characterizes the macroscopic transport efficiency of the foam, while the breakage rate reflects its microscopic structural stability.

From an algorithm validation perspective, they pose a symmetrical challenge: an algorithm that cannot accurately quantify breakage events (i.e., the disappearance of feature points) will likely produce unreliable velocity measurements, as it may misinterpret the disappearance of features as intense motion. Conversely, an algorithm unable to stably track features in high-speed motion cannot accurately assess the breakage rate. Therefore, traditional observation methods, which rely on manual experience, not only fail to quantify these intertwined key parameters but also suffer from issues like analytical lag and untimely control, severely constraining production efficiency.

Consequently, the adoption of machine vision for intelligent monitoring of the flotation process has become an industry trend. Previous studies have predicted flotation recovery rates by measuring foam characteristics [1,2], while recent research based on artificial intelligence, such as the use of such as You Only Look Once (YOLO) [3], has focused on developing systems for real-time extraction of foam parameters. It is worth noting that state-of-the-art machine learning (ML) approaches have seen rapid development. For instance, learned feature extractors like SuperPoint [4] and optical flow networks like Recurrent All-Pairs Field Transforms (RAFT) [5] offer impressive accuracy in general scenes, while generative models (e.g., Generative Adversarial Networks (GANs) [6]) show potential for synthesizing training data. However, applying these methods to flotation foam monitoring faces significant hurdles. First, acquiring pixel-level ground truth for dynamic motion vectors in foam is extremely labor-intensive compared to bounding-box annotations used in detection tasks (like YOLO). Although GANs can generate synthetic foam images, bridging the ‘domain gap’ to authentic, complex industrial textures remains a challenge. Second, flow network models are often computationally heavy, making them unsuitable for deployment on resource-constrained industrial edge devices requiring high-frequency monitoring. Third, ML models often operate as ‘black boxes’ with limited interpretability. Given these constraints, feature-based methods—which rely on explicit physical gradients and offer high interpretability—remain the optimal choice for industrial applications. Yet, they face their own set of challenges. While research on static foam image features has advanced, the extraction of dynamic features remains underdeveloped due to its complexity. Current studies predominantly focus on using static image features (e.g., foam size, color, texture) to predict flotation indicators, but still face significant challenges in the real-time, precise extraction of dynamic features. The core challenge lies in performing efficient and accurate feature matching between consecutive image frames [7,8]. Flotation foam images exhibit complex characteristics such as weak textures, severe non-rigid deformations, and motion blur, which makes many traditional feature matching algorithms (e.g., Scale-Invariant Feature Transform (SIFT), Oriented FAST and Rotated BRIEF (ORB)) inapplicable. For instance, the FAST corners relied upon by the ORB algorithm [8,9,10] are difficult to detect stably on the monotonous texture of the foam surface, while the severe deformations from foam rupture and coalescence lead to numerous descriptor matching errors. At a deeper level, these algorithms expose a common methodological flaw: they generally lack a systematic, coarse-to-fine mechanism for rejecting mismatched pairs, leading to an intractable trade-off between efficiency and precision.

To address these challenges, this paper moves beyond treating feature matching as a single step and instead proposes an innovative “progressive” methodology, realized through a multi-algorithm fusion framework based on “AKAZE-GMS-PROSAC.” Confronting the aforementioned methodological flaw, we have designed and constructed a new progressive feature matching framework. The design philosophy of this framework is precisely to establish a systematic, coarse-to-fine mismatch rejection mechanism: it first ensures the quality of source features through “stable extraction,” then rapidly filters a large number of obvious errors via “efficient coarse screening,” and finally guarantees geometric consistency through “precise matching,” thus forming a complete processing chain.

To address the weak texture problem, we selected the AKAZE algorithm [11,12,13,14]. By constructing a non-linear scale-space, it better preserves image edge details and can extract robust feature points even in regions with subtle texture changes, providing high-quality initial data for the subsequent ‘purification’ stages.

To tackle computational efficiency and the issue of numerous mismatches, we introduce the GMS algorithm [15,16,17]. It leverages the prior knowledge that matched point pairs should exhibit similar motion in their neighborhoods to filter out vast quantities of clear mismatches with extremely high efficiency, acting as an efficient ‘coarse filter’ and preventing wasted computational resources in subsequent steps.

To ensure final accuracy, we employ the PROSAC algorithm [18]. Through a more intelligent, progressive sampling strategy, it performs geometric verification on the matches filtered by GMS. Compared to the traditional Random Sample Consensus (RANSAC) algorithm [19], it can find the optimal model with fewer iterations, precisely eliminating residual mismatches and completing the final ‘refinement’ step.

The main contributions of this paper are as follows:

(1) Proposed a “progressive” feature matching framework (AKAZE-GMS-PROSAC) specifically designed for flotation foam dynamic analysis, effectively overcoming the challenges of feature matching in scenes with weak textures and large deformations.
(2) Validated the framework’s significant superiority in displacement and velocity estimation accuracy through comprehensive comparative experiments on real-world industrial datasets against mainstream solutions like ORB-GMS-RANSAC and ORB-RANSAC.
(3) Implemented and demonstrated a method for analyzing dynamic parameters based on this framework. In calculating the breakage rate, a novel “foam lifetime” parameter was introduced to more accurately identify valid unmatched points, thereby optimizing the calculation model. The analysis of flotation foam velocity features validates the advantages of the proposed algorithm, providing reliable technical support for the intelligent closed-loop control of the flotation process.

## 2. A Progressive Framework for Extracting Dynamic Foam Characteristics

**Figure 1:**
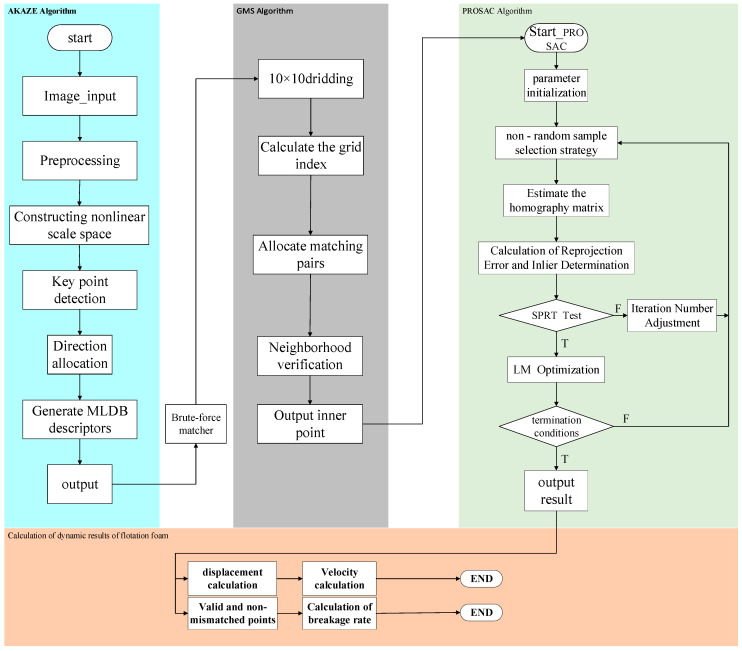
The algorithm principle block diagram.

To precisely extract the dynamic characteristics of flotation foam, this research devises a progressive technical pipeline termed “Stable Extraction—Efficient Coarse Screening—Precise Matching.” It is important to emphasize that this framework is not a simple, direct combination of standard algorithms, which often fail in flotation scenarios due to the “outlier avalanche” caused by repetitive foam patterns and weak textures. Instead, our pipeline is designed to efficiently and accurately eliminate mismatches through a synergistic, coarse-to-fine adaptation strategy. Figure 1 provides a visual representation of the complete workflow of this multi-algorithm fusion framework. Its core stages, along with their specific adaptations, are detailed as follows:

(1) Stable Extraction (Adapted AKAZE). To address the challenges of weak textures and severe deformations, this stage employs an adapted AKAZE algorithm. Unlike standard implementations, we optimized the diffusivity parameters and contrast thresholds of the non-linear scale-space. This allows the algorithm to specifically highlight bubble ridges in low-contrast regions while suppressing specular noise, providing high-quality, robust raw feature points for subsequent processing.
(2) Efficient Coarse Screening (Optimized GMS). The GMS algorithm is introduced as an efficient “coarse filter.” Crucially, we adapted the grid division scale (specifically optimizing it to a 10 × 10 grid, as detailed in Section 3.1.1) to align with the physical size of bubble clusters. Based on the principle of motion consistency, this optimized setting avoids disrupting the coherence of non-rigid foam motion and rapidly discards the vast majority of obvious mismatched pairs caused by repetitive patterns.
(3) Precise Matching (Targeted PROSAC). The PROSAC algorithm serves as a “fine filter.” It is synergistically adapted to operate solely on the high-confidence subset pre-filtered by GMS. Through rigorous progressive sampling and geometric constraint verification (with a stricter inlier threshold of 2.0 pixels), it functions as a rapid geometric refiner rather than a generic searcher, producing a final set of high-precision matches.

These high-precision matched pairs provide the critical data for the subsequent analysis of dynamic characteristics. Specifically, the calculation of these dynamic characteristics is twofold. First, the velocity of the foam is calculated by integrating the pixel displacement, scale factor, and frame interval information from all matched pairs. Second, the breakage rate is quantified by calculating the ratio of unmatched feature points to the total number of feature points in the current frame.

### 2.1. Dynamic Characteristic Parameters of Flotation Foam

The ultimate objective of this framework is to accurately calculate two key dynamic characteristics of flotation foam—velocity and breakage rate—based on stable and reliable feature matching results. Therefore, before elaborating on the specifics of the feature matching process, this section will first introduce the mathematical models used to calculate these two dynamic parameters. This serves to clearly define the framework’s output objectives and data requirements.

However, a critical challenge in defining these parameters is the non-rigid nature of flotation froth. The matching pipeline implicitly assumes geometric consistency, yet foam bubbles frequently undergo local stretching, shearing, and deformation. If not properly addressed, these non-rigid artifacts can be misidentified as breakage events or introduce bias into velocity estimation. To resolve this, our framework establishes a “Coarse-to-Fine Spatiotemporal Screening” strategy to strictly distinguish between non-rigid deformation and actual physical changes, ensuring the accuracy of both parameters:

(1) Impact on Breakage Rate: Distinguishing Deformation from Rupture via “Foam Lifetime” To distinguish non-rigid deformation from true breakage, we introduce a temporal parameter termed “Foam Lifetime” (defined in Section 2.1.2). The logic is as follows: The AKAZE descriptors used in our framework are constructed in a non-linear scale space, providing inherent robustness to local affine transformations. Consequently, when a bubble undergoes non-rigid deformation (stretching/shearing), its feature points remain matchable, maintaining their temporal continuity (increasing lifetime). In contrast, actual breakage involves a topological collapse of texture, rendering the feature unmatchable and resetting the lifetime. This mechanism ensures that the calculated breakage rate specifically reflects structural instability, filtering out artifacts caused by geometric deformation.
(2) Impact on Velocity: Filtering Non-Rigid Artifacts via Spatial Constraint Non-rigid deformation can produce erratic motion vectors that deviate from the bulk flow. To mitigate the impact of these artifacts on velocity estimation, we rely on the Spatial Screening of the PROSAC algorithm (Section 2.4). By enforcing a global geometric constraint (Homography), PROSAC treats the inconsistent vectors resulting from local deformation as outliers. This ensures that the computed velocity field represents the dominant physical advection of the stable foam, effectively eliminating the bias introduced by local non-rigid fluctuations.
(3) Spatiotemporal Coarse-to-Fine Rejection: By integrating the above mechanisms, the framework achieves a comprehensive rejection strategy. Spatial Domain (Coarse-to-Fine): GMS and PROSAC filter out geometric outliers within a single frame. Temporal Domain: The “Foam Lifetime” threshold filters out transient noise that lacks continuity across frames. Only features that demonstrate high stability in both spatial geometry and temporal tracking are utilized for the final dynamic analysis.

#### 2.1.1. Calculation of Foam Velocity

Based on the more precise feature point pairs obtained from the framework, the velocity of the foam can be quantified.

(1) Calculating Pixel Displacement

For the i-th matched pair, let the initial coordinates be $\left(x_{\mathrm{start}},y_{\mathrm{start}}\right)$ and the final coordinates be $\left(x_{\mathrm{end}},y_{\mathrm{end}}\right)$. The horizontal component of its displacement is then calculated:

(1) $$\begin{array}{c} D_{X}=x_{\mathrm{start}}-x_{\mathrm{end}} \end{array}$$

Vertical component:

(2) $$\begin{array}{c} D_{Y}=y_{\mathrm{start}}-y_{\mathrm{end}} \end{array}$$

(2) Calculating the Scale Factor

After obtaining the horizontal and vertical components, these pixel-based values must be converted to physical units. This is accomplished using the known camera specifications and the on-site imaging parameters through the principles of optical imaging. First, the scale factor, Q, is calculated:

(3) $$\begin{array}{c} Q=\frac{h_{\mathrm{img}}}{\mathrm{pix}} \end{array}$$

Here, $h_{\mathrm{img}}$ represents the imaging height of the sensor. pix denotes the total number of pixels in the image along this direction.

(3) Solve for instantaneous velocity:

Combining pixel displacement and scale factor, the instantaneous velocity v_i_ of the bubble microelement represented by the i-th matching pair is:

(4) $$v_{i}=\sqrt{D^{2}+D_{j}^{2}}QT$$

Here, T represents the time interval between image frames.

Finally, by averaging the velocities of n valid feature matching pairs, the overall average movement velocity $v_{\varepsilon }$ of the bubble at the current time can be obtained:

(5) $$\begin{array}{c} v_{\varepsilon }=\frac{1}{n}\sum_{i=1}^{n}v_{i} \end{array}$$

#### 2.1.2. Calculation of Foam Breakage Rate

The breakage rate is a critical dynamic indicator of foam stability. An ideal breakage rate should accurately reflect the breakage events of stable foam, rather than artifacts caused by image noise or transient, minor foam structures. To achieve this objective, this study introduces a “foam lifetime” parameter to enhance the robustness of the calculation.

“Foam lifetime” is an integer attribute assigned to each individual feature point, which is considered a representation of a foam bubble or a stable part of its structure. It quantifies the number of consecutive frames for which a feature point has been successfully tracked and matched since its initial detection. By establishing a minimum lifetime threshold, we can effectively differentiate between the actual breakage of stable foam and interference from detection noise.

Let A_t_(p_i_) denote the lifetime value of a feature point p_i_ at frame t. This value is updated using the following iterative formula:

(6) $$\begin{array}{c} A_{i}(p_{i})=\{\begin{array}{cc} A_{i-1}(p_{i})+1 & \mathrm{if}\;M(p_{i},t)=\mathrm{true}\\ 0 & \mathrm{if}\;M(p_{i},t)=\mathrm{false} \end{array} \end{array}$$

Here, t is the index of the current frame, p_i_ is a feature point from the preceding frame, and A_t−1_(p_i_) is its lifetime value in that preceding frame. M(p_i_,t) is a Boolean function representing the matching status, which returns true if p_i_ is successfully matched in the current frame. The initial lifetime for newly detected feature points is 0.

Building upon the “foam lifetime” concept, we define an “effective breakage point” as a feature point that existed in the preceding frame but fails to match in the current frame, provided its lifetime in the preceding frame has reached the predefined minimum threshold, T_life_.

This definition allows for the precise calculation of E, the number of effective breakage points in the current frame. Finally, the Breakage Rate is defined as the ratio of E, the number of effective breakage points, to A, the total number of feature points in the preceding frame, as shown in Equation (7):

(7) $$\begin{array}{c} Breakup.\;rate=\frac{E}{A}\times 100\% \end{array}$$

In summary, the breakage rate calculation method proposed in this study introduces the “foam lifetime” parameter to rigorously define an “effective breakage point.” A point is classified as an effective breakage only if it existed in the previous frame, its lifetime met the specified threshold, and it could not be matched in the current frame. This approach effectively filters out spurious breakage events caused by transient noise or minor occlusions. Ultimately, the breakage rate calculated via Equation (7) provides a more authentic reflection of the foam’s macroscopic stability.

### 2.2. Extraction and Description of Flotation Froth Image Feature Points Based on the AKAZE Algorithm

Based on the preceding description, and to address the challenges commonly present in flotation foam images, such as weak textures, uneven illumination, and non-rigid deformations, this paper employs the Accelerated-KAZE (AKAZE) algorithm for feature point extraction and description. This algorithm is characterized by its strong robustness, high feature distinctiveness, and adaptability to complex scenes. In contrast to algorithms such as SIFT, which depend on linear Gaussian scale-space, AKAZE constructs a non-linear scale-space (governed by the Perona–Malik equation). This approach more effectively preserves edge details between bubbles while simultaneously smoothing noise, a capability that is crucial for the stable detection of keypoints on the texture-scarce foam surface. The process is mathematically described by Equation (8):

(8) $$\begin{array}{c} \frac{\mathrm{dt}}{\mathrm{dT}}=div(g(|\nabla I|)\nabla I) \end{array}$$

In this equation, L(x,y,t) represents the image intensity at position (x,y) and scale t. It is important to note that the time variable t here corresponds to the scale parameter within the scale-space. The term ∇I is the gradient vector of the image, as defined previously in Equation (9).

(9) $$\begin{array}{c} \nabla I=(\frac{dx}{dI},\frac{dy}{dI}) \end{array}$$

Here, |∇I| is the magnitude of the gradient. The term g(|∇I|) is the diffusivity function, which typically takes the form of Equation (10).

(10) $$g(s)=\frac{1}{1+(\frac{s}{k}{)}^{2}}$$

In this equation, k is a positive constant that controls the level of diffusion. Unlike standard implementations where k is fixed, in this framework, we optimized the contrast factor k to a lower value. This adaptation enhances the algorithm’s sensitivity to subtle gradients, allowing for the superior preservation of faint bubble edges in weak-texture regions and exhibiting greater stability, particularly in noisy images. The algorithm detects feature points using the Hessian matrix, which is defined as shown in Equation (11).

(11) $$H=\left(\begin{array}{c} \begin{array}{cc} \frac{\partial^{2}I}{\partial x^{2}} & \frac{\partial^{2}I}{\partial x\partial y}\\ \frac{\partial^{2}I}{\partial y\partial x} & \frac{\partial^{2}I}{\partial y^{2}} \end{array} \end{array}\right)$$

The Hessian matrix is used to identify potential feature points by calculating its determinant (det(H)) and trace (tr(H)). When the det(H) exceeds a certain threshold, the point is classified as a feature point. To address the scarcity of texture on smooth bubble surfaces, we adjusted this detection threshold to be more permissive than in general scenarios. This strategy maximizes the recall of potential keypoints (bubble ridges), relying on the subsequent GMS stage to filter out any resulting noise. Due to the algorithm’s inherent multi-scale adaptability, the feature detection exhibits greater robustness to changes in scale and rotation.

The AKAZE feature descriptor is an evolution of the DAISY descriptor and multi-scale local binary patterns. Taking the Modified Local Difference Binary (MLDB) descriptor as an example, it is a binary descriptor that generates a binary code by comparing the intensity differences between a feature point and the pixels in its local neighborhood. For a feature point p and a neighboring pixel q_i_, the intensity difference between them is defined as shown in Equation (12). In contrast to the BRIEF descriptor used in the ORB algorithm, MLDB incorporates scale and rotation information during its construction. This makes the descriptor more robust to the scale changes and rotational shifts that foam undergoes during motion, thereby enhancing the accuracy of subsequent feature matching.

(12) $$\begin{array}{c} \Delta I_{i}=I(q_{i})-I(p) \end{array}$$

Subsequently, a binary bit is generated based on the sign of the value from Equation (12). Finally, these bits are combined into a binary vector to serve as the feature descriptor.

This descriptor can more effectively discriminate between similar features in complex scenes. It is precisely because the MLDB descriptor integrates scale and rotation information during its construction that it exhibits superior robustness to the morphological changes and rotations of the flotation foam during motion. This, in turn, provides a reliable foundation for achieving high-precision feature matching in the subsequent stages.

### 2.3. Rapid Filtering Based on Motion Consistency with GMS

The core assumption of the Grid-based Motion Statistics (GMS) algorithm is highly consistent with the physical motion characteristics of flotation foam: on a macroscopic scale, adjacent groups of foam bubbles typically move as a cohesive unit, exhibiting similar motion vectors. Consequently, for a correct matched pair, other correct matches within its surrounding neighborhood should also demonstrate similar motion directions and magnitudes. Any matched points whose motion patterns are incongruent with their neighborhood are, with high probability, mismatches.

**Figure 2:**
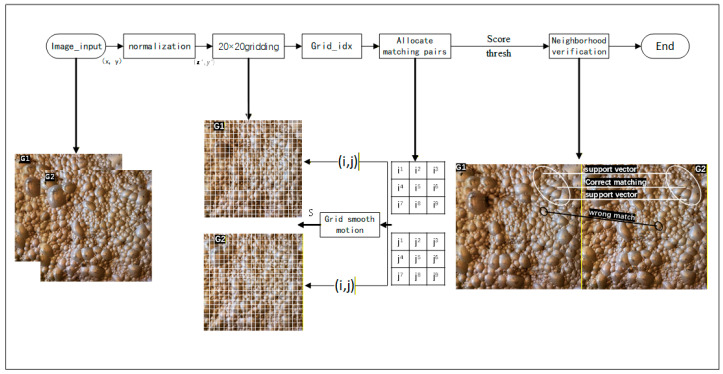
GMS inlier screening flow chart. The G1 and G2 are two consecutive frames. The white circle and black circle areas represent the correctly matched region and incorrectly matched region, respectively. The ends of the connecting lines indicate the matching points.

It is based on this principle that GMS can efficiently identify and reject these isolated mismatches whose motion patterns are inconsistent with their surrounding neighbors. The GMS algorithm screens for inliers by dividing the image into a grid and statistically analyzing the motion patterns of the matched points. A flowchart of the overall process is shown in Figure 2.

First, the coordinates of the keypoints are normalized to the interval to eliminate the influence of image size variations. The updated horizontal and vertical coordinates, denoted as x′ and y′, respectively, are calculated as shown in Equation (13).

(13) $$\begin{array}{c} x^{\prime }=\frac{x}{w},y^{\prime }=\frac{y}{h} \end{array}$$

The method for calculating grid indices involves partitioning the image into a grid of a fixed size (e.g., 20 × 20) and assigning each keypoint to its corresponding cell. The process for this calculation is shown in Equation (14).

(14) $$\begin{array}{c} grid\_dx=[x^{\prime }\times w]+[y^{\prime }\times h]\times w \end{array}$$

Here, w and h represent the width and height of the image grid, respectively. Subsequently, each matched pair is assigned to its corresponding grid cells in the left and right images. The grid indices for the left and right grids, (G_1_, G_2_), are calculated via Equation (14), and the number of matches for each grid pair ($g_{1i}$, $g_{2i}$) is tallied and stored in the motion matrix S, as shown in Equation (15).

(15) $$\begin{array}{c} S[g_{1i}][g_{2i}]=\sum j=ln\delta (g_{1j}-g_{1i})\delta (g_{2j}-g_{2i}) \end{array}$$

Here, δ(k) is the Kronecker delta function. This method effectively assigns matched pairs to their respective grid pairs, providing the basis for the subsequent neighborhood verification. During the neighborhood verification stage, for each grid cell (g_1_) in the left image and its corresponding grid cell (g_2_) in the right image, we calculate a match score based on its 3 × 3 neighborhood, as shown in Equation (16).

(16) $$\begin{array}{c} score=\sum_{k=1}^{9}=S[N_{1}(g_{1})[k]][N_{2}(g_{2})[k]] \end{array}$$

Here, N_1_(g_1_) and N_2_(g_2_) represent the sets of neighboring cells for grid points in the left and right images, respectively. To determine the validity of the match, a neighborhood verification threshold, thresh, is calculated as shown in Equation (17). In this equation, α is an empirical coefficient used to balance the stringency of the verification, n[N_1_(g_1_)[k]] is the number of keypoints within a neighboring grid cell in the left image, and nvalid is the number of valid neighboring grid pairs. If score > thresh, the grid pair is considered a valid match, and the corresponding feature pairs within it are classified as inliers. This process enables highly efficient inlier screening.

(17) $$\mathrm{thresh}=\alpha \times \sqrt{\frac{\sum_{k=1}^{9}n_{1}[N_{1}(g_{1})[k]}{nvalid}}$$

### 2.4. Progressive Geometric Verification Based on PROSAC

Although the GMS algorithm has performed an efficient ‘coarse screening,’ a final geometric verification is still required to eliminate more subtle mismatches (outliers). To this end, the framework incorporates the Progressive Sample and Consensus (PROSAC) algorithm as the final ‘precise matching’ stage. The core innovation of PROSAC lies in its intelligent, progressive sampling strategy. It discards the random sampling of traditional RANSAC and instead leverages a key insight regarding the non-uniform quality of matches filtered by GMS: the higher the score of a matched pair, the greater its probability of being an ‘inlier’.

Based on this principle, PROSAC preferentially samples from subsets of high-quality matches to estimate the geometric model (e.g., a homography matrix) and combines this with the Sequential Probability Ratio Test (SPRT) to make rapid decisions. This enables the algorithm to quickly converge to an optimal model composed of a ‘pure’ set of inliers in a minimal number of iterations.

**Figure 3:**
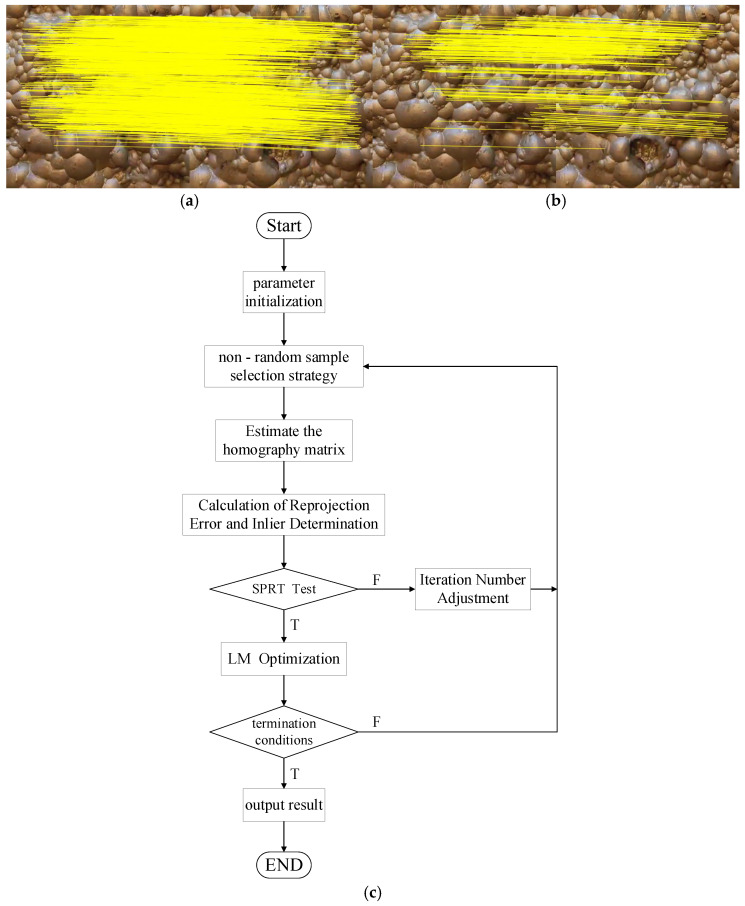
(**a**,**b**) feature matching; (**c**) PROSAC geometric verification algorithm flow chart. The yellow lines represent the connections between matching endpoints.

This rigorous geometric refinement process, while effectively filtering out mismatches, will also remove some ‘inliers’ that do not perfectly conform to the final model. Consequently, the total number of matched pairs is significantly reduced. This effect is visually demonstrated in Figure 3: the 1117 initial matches produced by GMS (Figure 3a) are ultimately refined by PROSAC into 253 high-confidence pairs (Figure 3b). The detailed principles of the PROSAC algorithm will be elaborated below, with its overall workflow illustrated in Figure 3c.

(1) Initial Estimation of the Homography Matrix

When addressing the image matching problem, estimating the homography matrix, H, is a necessary step to determine the geometric transformation between the two images. This matrix enables the projection of points from the source image onto the plane of the target image, as formulated in Equation (18).

(18) $$H\left(\begin{array}{ccc} h_{1} & h_{2} & h_{3}\\ h_{21} & h_{22} & h_{23}\\ h_{31} & h_{32} & 1 \end{array}\right)$$

(2) Calculation of Reprojection Error and Inlier Determination

Once the homography matrix H has been estimated, the reprojection error for each matched pair is calculated according to Equation (19). If the error is below a predefined threshold, maxD2, the pair is classified as an inlier. The objective of this step is to filter for the set of matches that are geometrically consistent with the current homography matrix. This process simultaneously rejects mismatches caused by noise and descriptor errors, as well as any geometrically correct matches that are inconsistent with the current model.

(19) $$\begin{array}{c} reproj{\mathrm{Dist}}_{i}={\left(\frac{h_{11}x_{i}+h_{12}y_{i}+h_{3}}{h_{31}x_{i}+h_{32}y_{i}+1}-X_{i}\right)}^{2}+{\left(\frac{h_{21}x_{i}+h_{22}y_{i}+h_{23}}{h_{31}x_{i}+h_{32}y_{i}+1}-Y_{i}\right)}^{2} \end{array}$$

(3) Sequential Probability Ratio Test (SPRT)

After an initial set of inliers is identified, the Sequential Probability Ratio Test (SPRT) is used to dynamically assess the validity of the current homography matrix. In this hypothesis testing framework, SPRT makes a dynamic decision based on the likelihood ratio defined in Equation (20). Here, P(D|H_1_) represents the probability of observing the current data under the alternative hypothesis (H_1_), which posits that the homography matrix is incorrect, leading to a high rate of mismatches. Conversely, P(D|H_0_) represents the probability of observing the data under the null hypothesis (H_0_), which posits that the homography matrix is correct, resulting in a high inlier ratio.

(20) $$\begin{array}{c} \begin{array}{c} \lambda =\frac{P(D|H_{1})}{P(D|H_{0})} \end{array} \end{array}$$

(4) Adjustment of the Iteration Count

To balance model accuracy with computational efficiency, the algorithm dynamically adjusts the required number of iterations. This adjustment is guided by the current inlier ratio and the target confidence level, with the specific calculation performed using the confidence cfd, the inlier ratio w, and the formulas presented in (21) and (22). Here, s represents the minimum number of sample points required to generate the model (s = 4 for homography estimation), while maxI is used to cap the maximum number of iterations and prevent infinite loops.

Concurrently, the algorithm employs a non-random sampling strategy (the N-R strategy), which preferentially selects high-confidence feature points for sampling. By integrating this strategy with the formula for adjusting the iteration count, the algorithm can effectively reduce the number of unproductive iterations, thereby significantly improving its runtime efficiency.

(21) $$\begin{array}{c} k=min(\frac{log(1-cfd)}{log(1-w^{5})},maxI) \end{array}$$

(22) $$\begin{array}{c} w=\frac{\mathrm{The}\;\mathrm{current}\;\mathrm{number}\;\mathrm{of}\;\mathrm{interior}\;\mathrm{points}}{\mathrm{The}\;\mathrm{total}\;\mathrm{number}\;\mathrm{of}\;\mathrm{interior}\;\mathrm{points}} \end{array}$$

(5) Levenberg—Marquardt (LM) Optimization

Finally, the Levenberg–Marquardt (LM) optimization algorithm is employed to minimize the objective function. The objective function, S, is defined as the sum of the squared reprojection errors for all inliers, as shown in Equation (23). (The reprojection error is calculated using Equation (19)).

After an initial homography matrix is obtained using a random sampling consensus algorithm like RANSAC, it is typically not the optimal solution. The LM algorithm can further refine this initial estimate by minimizing the sum of the squared reprojection errors to improve the model’s accuracy. Its fundamental principle is to iteratively update the model parameters by combining the Jacobian matrix, J, with an approximation of the Hessian matrix, $J^{T}J$. The specific update process is detailed in Equation (24), diag is a mathematical operator representing a diagonal matrix.

(23) $$S=\sum_{i=1}^{N}{{\mathrm{reprojDist}}_{i}}^{2}$$

(24) $$(H+\Delta H)=H-(J^{T}J+\lambda \mathrm{diag}(J^{T}J){)}^{-1}J^{T}e$$

Thus, through the rigorous progressive sampling of the PROSAC algorithm, the Sequential Probability Ratio Test, and the final Levenberg–Marquardt optimization, the last ‘precise matching’ stage of the feature matching process is completed. This series of steps ensures that from the set of matches filtered by GMS, a geometrically highly consistent set of inliers can be extracted, yielding the optimal transformation model.

Through the complete workflow described above, from stable extraction (AKAZE), to efficient coarse screening (GMS), and finally to precise matching (PROSAC), this paper establishes a robust, progressive framework for dynamic feature matching. This framework can effectively and stably extract high-precision feature matches from flotation foam images. The following sections will present a series of experiments to comprehensively validate the performance and practical application of this framework.

## 3. Experiments and Application Validation

To validate the feasibility and superior performance of the “progressive” feature matching framework proposed in this paper within real-world industrial scenarios, a series of experiments were conducted using video imagery from a copper-lead mineral flotation plant. This chapter will first introduce the overall experimental design and evaluation methodologies. Subsequently, the performance of the framework will be validated through a comparative analysis against mainstream approaches, including an in-depth analysis of its underlying mechanisms. Finally, we will demonstrate how the framework is utilized to precisely calculate key dynamic characteristics, such as foam velocity and breakage rate.

### 3.1. Experimental Setup and Parameter Optimization

To comprehensively evaluate the algorithm’s performance, eight sets of flotation foam video sequences (T1–T8) were collected from a copper-lead flotation plant, representing a wide range of operating conditions from stable flow to high-turbulence bursting. The dataset parameters are as follows, Resolution: 500 × 500 pixels, Frame Rate: 15 fps, Ground Truth: Manually annotated displacement vectors for 100 bubbles per condition. Clarification on Units: It is crucial to note that the displacement and velocity values reported in the subsequent analysis (including Table 1 and Table 2) are measured in pixels and pixels/second, respectively, unless otherwise stated. To convert these values to physical velocity (m/s), the Scale Factor (Q) defined in Equation (3) must be applied. For instance, the high velocity observed in scenario T6 (approx. 776 pixels/s) corresponds to a physical speed of approximately 1.35 m/s (776 × 0.001746), which is consistent with actual industrial conditions. These image sets were processed for comparative analysis using the ORB-GMS-RANSAC algorithm, the ORB-RANSAC algorithm, and the framework proposed in this paper.

The test system was configured with a 13th Gen Intel^®^ Core™ i5-13400F processor (Intel Corporation, Santa Clara, CA, USA), an NVIDIA GeForce RTX 5060 graphics card (NVIDIA Corporation, Santa Clara, CA, USA), and was run on the Windows 10 operating system (Microsoft Corporation, Redmond, WA, USA). The proposed algorithm was implemented in C++ using Visual Studio 2022, offering both high portability and computational speed, making it suitable for complex industrial flotation scenarios.

To thoroughly assess the framework’s performance, this chapter outlines a two-stage experimental process with a logical progression. The first stage consists of parameter optimization experiments, aimed at identifying the optimal combination of key internal parameters to establish a fair baseline for subsequent comparisons. The second stage involves performance comparison experiments, where under these optimal parameters, our framework is subjected to rigorous quantitative and qualitative comparisons against mainstream algorithms.

#### 3.1.1. Parameter Optimization and Adaptation Experiments

The performance of the proposed algorithm is significantly affected by two key parameters: the grid size G of the GMS algorithm and the inlier threshold of the PROSAC algorithm. These parameters require specific adaptation to the flotation environment, which involves dense, repetitive patterns and non-rigid motion. A smaller G value (denser grid) typically leads to finer motion granularity but increases computational load, whereas an overly large grid may fail to capture the local motion coherence of small bubble clusters. Similarly, the PROSAC threshold must be carefully tuned: a lower value enforces a stricter filtering criterion, which increases runtime and reduces the final count of matched feature points, but if set too low, it may reject valid features undergoing slight non-rigid deformation. Due to the coupled influence of these two parameters, we employed a controlled variable methodology to optimize them in a stepwise manner.

**Figure 4:**
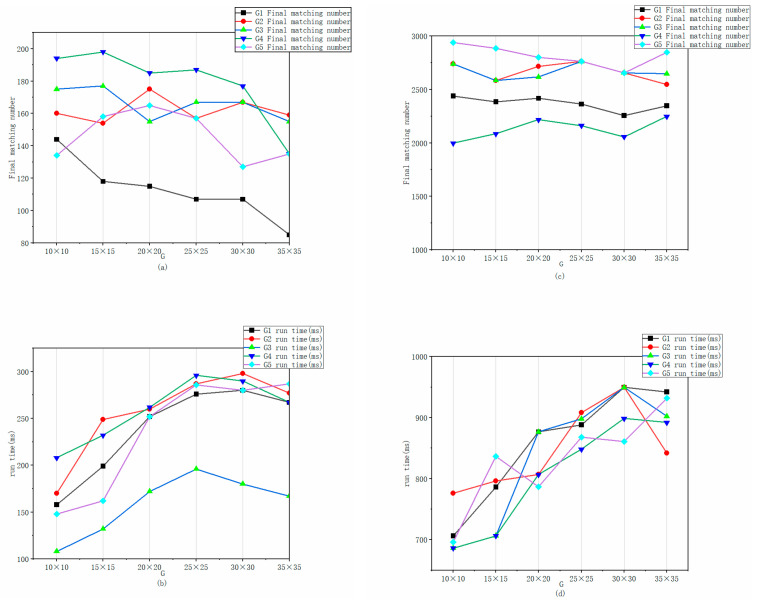
Comparison results when different grid sizes G are taken with the PROSAC threshold fixed at 3 in the algorithm of this paper. (**a**) The fixed threshold algorithm takes different grids G for the corresponding matching points of froth velocity measurement; (**b**) The fixed threshold algorithm takes different grids G to measure the corresponding running time of the froth velocity; (**c**) The fixed threshold algorithm takes different grids G to measure the corresponding matching points of the froth breakage rate; (**d**) The fixed threshold algorithm takes different grids G to measure the corresponding running time of the froth breakage rate.

First, to evaluate the impact of the grid size G, the PROSAC threshold was fixed at an empirically chosen intermediate value of 3. The influence of different grid sizes was then systematically tested to determine the optimal G value. As shown in Figure 4, with the PROSAC threshold fixed at 3 for the foam velocity measurement scenario, the runtime of the proposed algorithm fluctuates but shows a distinct efficiency peak at a grid size of 10 × 10. Concurrently, the number of matched feature points exhibits a stable downward trend as the grid size increases. Crucially, we observed that a grid size of 10 × 10 yields the optimal balance between speed and accuracy. At this scale, the grid division aligns well with the physical spatial distribution of bubble clusters, allowing the algorithm to effectively filter out mismatches caused by repetitive patterns without disrupting the coherent motion of non-rigid foam. Therefore, a 10 × 10 grid was selected as the optimal size for this study.

Following the grid size selection, we further optimized the PROSAC inlier threshold. Through iterative testing, we determined that a threshold of 2.0 pixels provides the highest accuracy. This value is strict enough to reject outliers caused by bubble rupture but loose enough to accommodate slight non-rigid deformations (e.g., stretching) between frames, ensuring robust tracking.

In summary, through the parameter optimization experiments described above, we have systematically determined the optimal parameter combination for our framework: a GMS grid size (G) of 10 × 10 and a PROSAC threshold of 2. This configuration is used in all subsequent comparative experiments to ensure a fair and valid performance evaluation.

#### 3.1.2. Performance Comparison Methodology

After establishing the optimal parameter combination, we proceeded to the performance comparison stage. The harsh environment of the flotation process—where foam deformation, breakage, and morphological changes can all affect algorithm performance—informed our dataset preparation. We directly utilized on-site imagery of flotation foam collected from the mining plant. This provides a real-world industrial dataset (the aforementioned T1–T8) that includes a wide variety of complex operating conditions, allowing for a comprehensive evaluation of our framework’s practicality and robustness.

**Figure 5:**
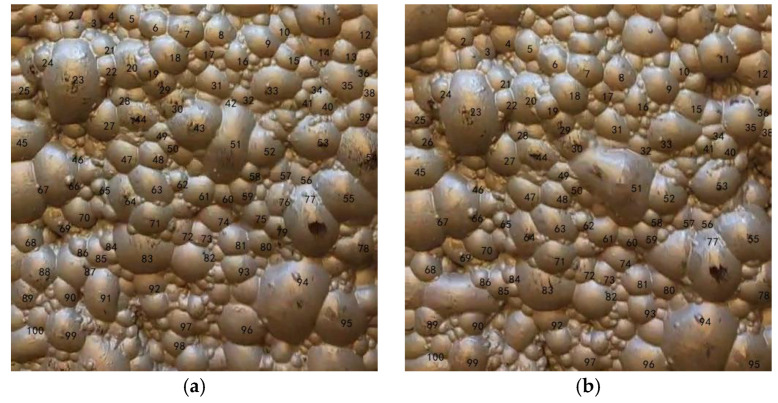
Manual annotation: (**a**) Annotation for the previous frame; (**b**) Annotation for the subsequent frame. The corresponding numbers in the images indicate the points that correspond to each other between the two images, which are the result of manual annotation.

Next, for ground truth annotation of the velocity parameter, we used the Label-Studio(v1.8.0) software to manually annotate the cross-frame displacement of 100 individual foam bubbles in each of the eight operating conditions. Each bubble was assigned a persistent ID. In the two image frames, the same identifier was used to mark the corresponding features of a single bubble, and the output for each label consisted of its horizontal and vertical coordinates. As shown in Figure 5. The output was generated in JSON format, and a subsequent script was used to de-normalize these coordinates to their original pixel dimensions. This annotation process was independently performed by three experts, and the results were cross-validated to ensure consistency. The final ground truth for foam velocity and displacement was determined by averaging the annotations.

#### 3.1.3. Evaluation Metrics

To quantitatively evaluate the advantages of the proposed algorithm, the selected evaluation metrics include Displacement Error and the Correct Match Ratio (CMR). The Signed Displacement Error (SDE) is defined as:

(25) $$\begin{array}{c} \mathrm{SDE}=({D\;}_{algorithm}-{D\;}_{Manually\;Annotated}) \end{array}$$

where D represents the magnitude of the displacement. A positive SDE indicates an overestimation by the algorithm, while a negative SDE indicates an underestimation. Similarly, the Relative Error (RE) preserves the sign to reflect the percentage bias:

(26) $$\begin{array}{c} \mathrm{RE}\;=\;(\mathrm{SDE}\;/\;\mathrm{Manual}\;\mathrm{Annotation})\;\times \;100\%) \end{array}$$

To summarize the overall accuracy of the entire dataset, we calculate the Mean Absolute Error (MAE) and Mean Relative Error (MRE). Note that to accurately reflect the magnitude of deviation, both MAE and MRE are computed using the absolute values of each error, as shown in the following formulas:

(27) $$\begin{array}{c} \mathrm{MAE}=\frac{1}{n}\sum_{i=1}^{n}|{\mathrm{SDE}}_{i}| \end{array}$$

(28) $$\begin{array}{c} \mathrm{MRE}=\frac{1}{n}\sum_{i=1}^{n}|RE_{i}| \end{array}$$

The Correct Match Ratio (CMR) is defined as:

(29) $$\begin{array}{c} \mathrm{CMR}=\frac{m_{c}}{m} \end{array}$$

where m_c_ is the number of correctly matched feature pairs, and m is the total number of matched feature pairs.

With this, the experimental datasets, ground truth benchmarks, and evaluation metrics required for the performance comparison have all been defined. The following section will build upon this foundation to conduct a comprehensive performance comparison and an in-depth mechanistic analysis, evaluating our framework against mainstream algorithms on the two core indicators of “displacement estimation accuracy” and “matching stability.”

### 3.2. Comparative Performance Against Mainstream Approaches

With the optimal parameters established (G = 10 × 10; PROSAC threshold = 2), we conducted rigorous comparative experiments to comprehensively evaluate the performance of our framework (AKAZE-GMS-PROSAC, hereinafter A-G-P) against two mainstream approaches: ORB-GMS-RANSAC (hereinafter O-G-R) and ORB-RANSAC (hereinafter O-R), across multiple real-world industrial scenarios.

**Figure 6:**
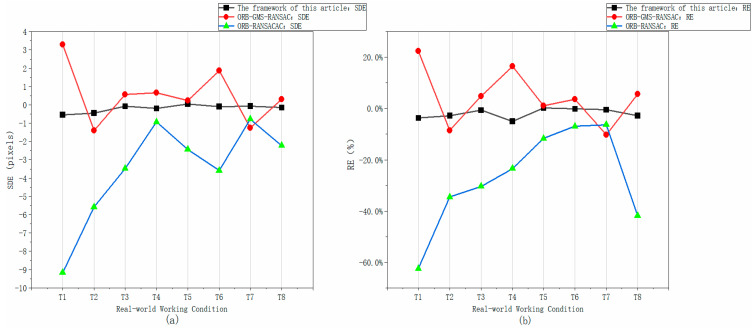
Comparison diagram of the relative errors of the three algorithms. (**a**) Comparison chart of SDE of the three algorithms; (**b**) Comparison chart of RE of the three algorithms.

First, the overall performance data reveals a clear and commanding superiority of the proposed framework. As shown in Table 2, the Mean Absolute Error (MAE) of our framework is 0.23 pixels with a Mean Relative Error (MRE) of 2.13%, which are significantly lower than those of O-G-R (MAE: 1.20 pixels; MRE: 9.10%) and O-R (MAE: 3.53 pixels; MRE: 27.36%). This advantage is further corroborated by the error distribution plots (Figure 6a,b). The error curve for our framework is not only the lowest in magnitude but also remains stable within a narrow band of ±0.5 pixels, demonstrating exceptional stability and robustness. In contrast, the error values for the two competing algorithms are not only higher but also fluctuate dramatically, indicating poor stability.

Furthermore, this significant performance advantage is rooted in the progressive optimization design mechanism of our framework. Unlike the competing algorithms, which rely on ORB features, the core advantage of our framework lies in its construction of a complete processing chain, from “stable extraction” to “progressive refinement”:

Stable Extraction: It employs the AKAZE algorithm, which is more robust to weak textures and scale changes, as the feature extractor. This ensures the quality of the feature points at the source.

Progressive Refinement: It combines the GMS algorithm for efficient ‘coarse screening’ based on motion consistency, which rapidly filters out a large number of obvious mismatches. This is followed by the use of the PROSAC algorithm’s progressive sampling strategy for ‘precise refinement,’ which accurately eliminates residual and more complex mismatches.

It is precisely this synergistic architecture that allows our framework to systematically overcome the performance bottlenecks that plague traditional methods in complex scenarios. Beyond algorithmic precision, a critical concern in foam velocimetry is ensuring that the estimated velocity reflects true physical advection rather than appearance artifacts caused by bubble rupture, coalescence, or illumination gradients. Our framework addresses this potential bias through two mechanisms. First, regarding feature selection, the AKAZE algorithm operates in a non-linear scale space. Unlike linear Gaussian blurring, this approach effectively diffuses high-frequency noise and transient lighting variations (such as specular highlights) while preserving the structural edges of bubble ridges. This ensures that the tracked keypoints represent stable physical structures rather than fleeting optical phenomena. Second, the PROSAC geometric verification serves as a robust filter against non-advective motion. Feature points affected by rupture or shifting illumination typically generate chaotic motion vectors that deviate from the coherent bulk flow. PROSAC identifies the dominant motion model (the consensus set) and classifies these inconsistent vectors as outliers, excluding them from the final velocity calculation. The validity of this approach is empirically supported by the results in Table 1, where the high consistency between our algorithm’s estimation and the manual ground truth (which tracks physical bubbles) confirms that the bias introduced by appearance features is negligible. Its specific advantages are especially prominent in the comparative analysis of the following typical scenarios.

(1) High-Displacement Scenario (e.g., T6, with a manually annotated displacement of 51.76 pixels): The ORB-GMS-RANSAC algorithm produced a Signed Displacement Error (SDE) of +1.88 pixels (with a Relative Error, RE of 3.63%), while the ORB-RANSAC SDE was as high as +3.59 pixels (RE: 6.93%). This positive bias indicates that ORB features are susceptible to interference from large-scale motion, leading to matching drift and model overfitting. In contrast, our framework benefits from the non-linear scale-invariance of AKAZE and the progressive, optimized fitting of PROSAC, which effectively mitigates these issues.
(2) Low-Displacement/Low-Texture Scenario (e.g., T4, with a manual annotation of 4.0 pixels): The ORB-GMS-RANSAC and ORB-RANSAC algorithms yielded SDE values of +0.66 pixels (RE: 16.5%) and +0.94 pixels (RE: 23.4%), respectively, whereas our framework’s RE was a low −5.00% (SDE: −0.20 pixels). This highlights that, under weak-texture conditions, the non-linear feature extraction capability of AKAZE is far superior to that of ORB. Furthermore, the two-stage filtering mechanism (GMS+PROSAC) ensures that a high-purity set of matches can be obtained even when features are sparse, thus avoiding the model failure issues common in traditional methods.

**Figure 7:**
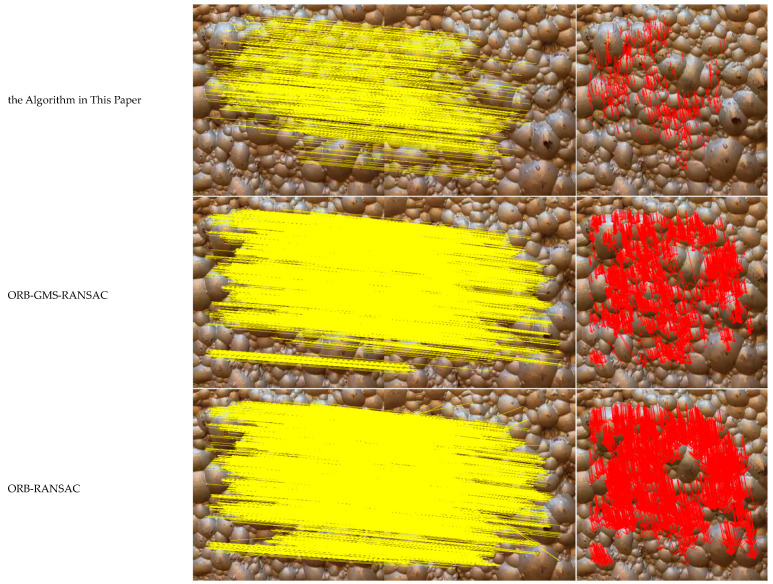
Comparison diagram of algorithm results under the T6 working condition. Yellow lines represent connections between feature matching points, while red arrows indicate velocity vectors.

The algorithm proposed in this paper, through its multi-scale feature extraction and progressive optimization strategies, achieves a robust performance breakthrough in both low-displacement/low-texture (T4) and high-displacement (T6) scenarios. This demonstrates its high precision and strong adaptability in complex engineering environments. A specific analysis of the high-displacement scenario T6 is presented in Figure 7. In the feature matching diagram generated by our proposed algorithm, no mismatches were observed, achieving a Correct Match Ratio (CMR) of 100%. The corresponding velocity vector field shows a distribution that is orderly and coherent, indicating that the algorithm accurately captures the displacement velocity information. This success is because the algorithm enhances scale robustness with AKAZE, rapidly eliminates mismatches with GMS, and optimizes the model fit with PROSAC. This combination effectively mitigates the problems of matching drift and model overfitting in high-displacement scenarios, resulting in a vector field that more closely aligns with the actual displacement velocity.

In comparison, the feature matching and velocity vector diagrams for the ORB-GMS-RANSAC algorithm appear overly complex relative to our framework. Its vector field exhibits a degree of disorder and deviation from the ground truth, with some vectors showing implausible directions and magnitudes. This is a direct result of the feature matching drift that occurs with the ORB algorithm under large-scale motion, which leads to errors in velocity estimation and demonstrates a lack of robustness compared to our proposed algorithm. As for the ORB-RANSAC algorithm, its feature matching process is plagued by a high volume of spurious matches, which significantly compromises the subsequent velocity measurements and causes the calculated velocity to deviate substantially from the actual velocity.

As can be clearly seen from the error analysis plots for the three algorithms presented in Figure 6a,b, the error of the framework proposed in this paper is the closest to the ground truth, in terms of both relative and absolute error. Compared to the other two algorithms, the curve corresponding to our method is also more stable and exhibits superior robustness.

In summary, these experiments demonstrate that the proposed framework achieves high precision and stability in displacement estimation, thereby establishing a solid foundation for the subsequent extraction of dynamic characteristics.

### 3.3. Application Validation: Analysis of Dynamic Characteristics

Velocity and breakage rate are the two key parameters that reflect the dynamic characteristics of flotation foam. The principles for their calculation were detailed in Section 2.1. This section will therefore analyze and calculate these dynamic characteristics of the flotation foam to validate the precise analytical capabilities of our framework.

(1) Calculation of Foam Breakage Rate

As shown in Table 3 below, the calculation results were obtained from video stream data with a frame interval of 1/15th of a second. After processing by the GMS-PROSAC algorithm, the number of reliable matched points is obtained. The GMS algorithm primarily performs a pre-screening during the feature matching stage to enhance matching efficiency and precision, while the PROSAC algorithm further refines the results during the geometric verification stage to address matching failures in complex scenarios. The synergistic effect of these two components ensures a more reliable and accurate foam analysis.

The number of unmatched feature points is calculated as: Total Features (Features 1)—Reliable Matches (PROSAC Matches). By incorporating the “foam lifetime” definition from the algorithm, the number of effective unmatched points can be determined. Dividing this by the total number of features yields the breakage rate. Table 3 presents a sample of the breakage rate calculation results.

(2) Calculation of Flotation Foam Velocity

Following the calculation principles detailed in Section 2.1.1, the process begins by acquiring a pair of consecutive foam images and performing an initial brute-force feature match. This initial set of matches is then filtered using the GMS algorithm to identify a set of probable inliers, which is subsequently refined through geometric verification with PROSAC to eliminate any remaining spurious matches.

**Figure 8:**
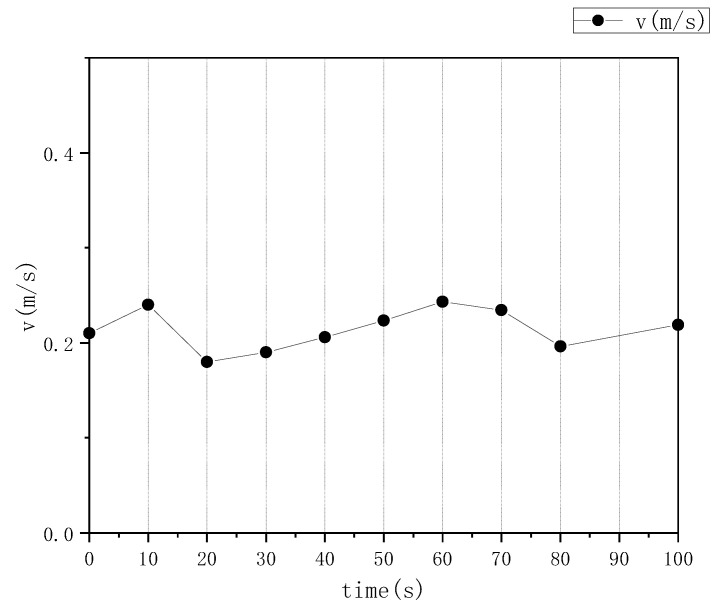
Distribution diagram of average flotation froth velocity.

With this high-purity set of matched pairs, the velocity calculation model from Section 2.1 is applied to determine the velocity magnitude and generate a velocity vector field. Finally, the actual velocity data (in m/s) is obtained by applying a scale factor that converts pixel displacements to real-world distances. For the on-site dataset used in this analysis, the frame interval is 1/15th of a second, and the image dimensions are 500 × 500 pixels. This configuration yields a scale factor (Q) of 0.001746 m per pixel. The average foam velocity was calculated over 10 s intervals, with the results presented in Figure 8.

## 4. Conclusions and Outlook

To address the inherent trade-off between efficiency and accuracy in extracting the dynamic characteristics of foam in industrial flotation, this paper has proposed and successfully validated an innovative “progressive” feature matching framework. Through a logically clear, progressively deepening pipeline of “stable extraction—efficient coarse screening—precise matching,” this framework systematically resolves the performance bottlenecks encountered by traditional algorithms in complex scenarios involving weak textures, severe deformations, and motion blur.

The success of this algorithm stems from the synergistic integration and collaborative effect of its three modules. First, the AKAZE algorithm, through its construction of a non-linear scale-space and robust feature extraction, accurately captures local foam features, providing high-quality input for the entire optimization workflow. Subsequently, the GMS algorithm acts as an efficient “coarse filter,” leveraging grid-based motion statistics to rapidly eliminate a vast number of mismatches, thereby dramatically enhancing matching efficiency. Finally, the PROSAC algorithm completes the “precise matching” stage by using geometric verification to further reject inconsistent matches and outliers. The synergistic fusion of these three components overcomes the limitations of traditional single-algorithm approaches in flotation foam image analysis, paving a new technical pathway for dynamic foam analysis.

This technology surmounts the performance bottlenecks of conventional methods in complex environments, particularly in scenarios with large displacements and motion blur, and significantly enhances the accuracy and real-time performance of dynamic characteristic analysis. Industrial validation confirms that this technology provides reliable technical support for the intelligent upgrading of the flotation process. Although the algorithm exhibited excellent performance in the current experiments, further optimization is still warranted:

Adaptive Parameter Mechanisms: Several parameters in the current algorithm (such as the GMS grid size and the PROSAC inlier threshold) are fixed. Future research could explore the integration of online or reinforcement learning mechanisms, which would enable the algorithm to dynamically adjust its parameters in real-time based on froth morphology (e.g., bubble size, flow velocity). This would achieve true adaptivity and enhance its resilience in variable operating conditions.

Multi-Modal Information Fusion: The dynamic information extracted by this framework, such as velocity and breakage rate, could be fused with morphological features derived from deep learning (e.g., bubble size distribution, stability). This would allow for the construction of a more comprehensive evaluation model for the flotation process, providing a richer basis for decision-making in the precise, closed-loop control of reagent dosage and aeration.

Edge Computing and Hardware Deployment: To meet the stringent real-time requirements of industrial sites, the algorithm model can be subjected to lightweighting and deployed on edge computing devices such as FPGAs or embedded GPUs. This would enable low-latency, localized, real-time monitoring and analysis of the flotation cell.

## Figures and Tables

**Table 1:** Comparison of the bubble velocity index obtained by three algorithms (units: displacement in pixels; velocity in pixels/s).

Real-World Working Condition	Manually Annotated Avg. D	Manually Annotated Avg. V	Avg. D: O-G-R	Avg. V: O-G-R	Avg. D: O-R	Avg. D: O-R	Avg. D: A-G-P	Avg. V: A-G-P
T1	14.67	220.00	17.95	269.25	23.83	357.50	14.00	211.88
T2	16.16	242.40	14.77	221.55	21.74	326.05	15.71	235.65
T3	11.44	171.60	12.00	180.00	14.92	223.78	11.37	170.49
T4	4.00	60.00	4.66	69.90	4.94	74.03	3.80	57.00
T5	20.97	314.51	21.17	317.55	23.40	351.00	21.12	316.80
T6	51.76	776.46	53.64	804.66	55.35	830.29	51.67	775.05
T7	12.23	183.39	10.98	164.63	13.00	195.00	12.16	182.45
T8	5.30	79.50	5.60	84.00	7.59	112.78	5.16	77.33

**Table 2:** Solution for the main performance comparison of three algorithms.

Real-World Working Condition	Manually Annotated Avg. D	O-G-R: SDE (pixels)	O-G-R: RE (%)	O-R: SDE (Pixels)	O-R:RE (%)	A-G-P: SDE (Pixels)	A-G-P: RE (%)
T1	14.67	3.28	22.36	9.16	62.44	−0.67	−4.57
T2	16.16	−1.39	−8.60	5.58	34.53	−0.45	−2.78
T3	11.44	0.56	4.90	3.48	30.42	−0.07	−0.61
T4	4.00	0.66	16.50	0.94	23.50	−0.20	−5.00
T5	20.97	0.20	0.95	2.43	11.59	0.15	0.72
T6	51.76	1.88	3.63	3.59	6.94	−0.09	−0.17
T7	12.23	−1.25	−10.22	0.77	6.30	−0.07	−0.57
T8	5.30	0.30	5.66	2.29	43.21	−0.14	−2.64
Mean Absolute Error (MAE/MRE)	-	1.20	9.10	3.53	27.36	0.23	2.13

**Table 3:** Some results of froth breakage rate.

Time (ms)	Features1	Features2	Raw	GMS	PROSAC	Breakup Rate (%)
701	6721	6771	6721	4441	1650	25.25
659	6612	6624	6612	5334	3260	23.31
627	6617	6617	6617	4604	1908	23.06
653	6616	6578	6616	5290	2628	22.01
650	6794	6670	6794	3738	1696	17.66
690	6729	6731	6729	5457	2298	16.14
601	6623	6570	6623	3896	1380	15.51
642	6581	6603	6581	5341	2232	13.90
695	6712	6735	6712	5907	2597	13.30
654	6825	6700	6825	4528	1596	13.03
657	6755	6808	6755	5238	2489	12.42
667	6591	6708	6591	4786	2157	12.41

## Data Availability

The data presented in this study are available on request from the corresponding author.

## Abbreviations

The following abbreviations are used in this manuscript:

AKAZE	(Accelerated-KAZE algorithm): The algorithm is an accelerated version of the KAZE algorithm for image feature detection and characterization.
GMS	(Grid-based Motion Statistics algorithm): The algorithm is an efficient filtering algorithm for image feature matching.
PROSAC	(PROgressive SAmple and Consensus algorithm): The algorithm is an optimized version of a stochastic parameter estimation method that is mainly used for feature matching and model fitting tasks in computer vision.
ORB	(Oriented FAST and Rotated BRIEF): The algorithm is an efficient image feature detection and description algorithm widely used in real-time computer vision tasks.
RANSAC	(Random Sample Consensus): The algorithm is a robust algorithm for estimating the parameters of a mathematical model through iteration.
